# Evaluation of anidulafungin in the treatment of intra-abdominal candidiasis: a pooled analysis of patient-level data from 5 prospective studies

**DOI:** 10.1007/s10096-019-03617-9

**Published:** 2019-07-06

**Authors:** Gabriele Sganga, Minggui Wang, M. Rita Capparella, Margaret Tawadrous, Jean L. Yan, Jalal A. Aram, Philippe Montravers

**Affiliations:** 1 Emergency Surgery, Fondazione Policlinico Universitario A. Gemelli IRCCS, Università Cattolica del Sacro Cuore, Largo A. Gemelli 8, 00168 Rome, Italy; 2 Huashan Hospital, Fudan University, Shanghai, China; 3 Pfizer PIO, Paris, France; 4 Pfizer Inc, Groton, CT USA; 5 Pfizer Inc, Collegeville, PA USA; 6 Paris Diderot Sorbonne Cité University and Bichat-Claude Bernard University Hospital, Paris, France

**Keywords:** Anidulafungin, Intra-abdominal candidiasis, Pooled analysis, Patient-level data, Efficacy, Safety

## Abstract

The incidence of nosocomial invasive fungal infections involving *Candida* spp. has increased markedly in recent years in patients undergoing abdominal surgery. This post hoc analysis aimed to determine the efficacy and safety of anidulafungin treatment in patients with intra-abdominal candidiasis (IAC) from five prospective studies (one comparative and four open-label) of adult surgical patients with microbiologically confirmed *Candida* intra-abdominal infection. Patients received an intravenous (IV) loading dose of anidulafungin 200 mg, followed by a daily 100-mg maintenance dose. Per study protocols, some patients could be switched to an oral azole after ≥ 5 or ≥ 10 days of IV treatment. Antifungal treatment was maintained for ≥ 14 days after the last positive *Candida* culture and resolution of symptoms. The global response rate (GRR) at the end of IV treatment (EOIVT) was the primary endpoint. GRR at the end of therapy (EOT), all-cause mortality at days 14 and 28, and safety was also evaluated. Seventy-nine patients had IAC from peritoneal fluid or hepatobiliary tract. *C. albicans* (72.2%) and *C. glabrata* (32.9%) were the most common pathogens. Overall GRR was 73.4% and 67.1% at EOIVT and EOT, respectively. All-cause mortality was 17.7% at day 14 and 24.1% at day 28 in the modified intent-to-treat population. Anidulafungin was well tolerated in this population, with most adverse events mild or moderate in severity. In these patients with IAC, anidulafungin showed a GRR at EOIVT similar to the anidulafungin registrational trial, and the results of our analysis confirmed the known safety profile of anidulafungin. ClinicalTrials.gov registration number NCT00496197, registered July 3, 2007, https://clinicaltrials.gov/ct2/show/study/NCT00496197; ClinicalTrials.gov registration number NCT00548262, registered October 19, 2007, https://clinicaltrials.gov/ct2/show/record/NCT00548262; ClinicalTrials.gov registration number NCT00537329, registered September 25, 2007, https://clinicaltrials.gov/ct2/show/record/NCT00537329; ClinicalTrials.gov registration number NCT00689338, registered May 29, 2008, https://clinicaltrials.gov/ct2/show/study/NCT00689338; ClinicalTrials.gov registration number NCT00805740, registered November 26, 2008, https://clinicaltrials.gov/ct2/show/NCT00805740

**Electronic supplementary material:**

The online version of this article (10.1007/s10096-019-03617-9) contains supplementary material, which is available to authorized users.

## Introduction

The incidence of nosocomial invasive fungal infections involving *Candida* species has increased in recent years because of the increasing number of immunocompromised patients, including patients with cancer, patients undergoing transplants, patients with human immunodeficiency virus and patients in intensive care units (ICUs) [1–4].

Clinical data on intra-abdominal candidiasis (IAC) are still scarce, although IAC is a common type of invasive candidiasis following candidaemia [5]. IAC is confirmed in patients when (i) there is clinical evidence of intra-abdominal infection and (ii) *Candida* isolates are collected from an intra-abdominal site under sterile conditions within 24 h [5, 6]. IAC, or *Candida*-associated peritonitis, is a common cause of mortality in patients in the ICU [1, 7, 8]. Previous studies have demonstrated the predominance of *C. albicans* isolates (approximately 75%), followed by *C. glabrata*, in intra-abdominal *Candida* infections in surgical patients [9, 10].

High rates of non-albicans *Candida* isolates from abdominal samples have been reported in patients in the ICU [7, 11]. In patients admitted to ICUs with post-operative peritonitis, multidrug-resistant strains were frequent and increased with the number of reoperations for persistent abdominal sepsis [12]. In a study of patients with post-operative peritonitis following bariatric surgery, 25 out of 61 patients were positive for *Candida* infections (6 [22%] were fluconazole-resistant *C. glabrata*) and 36 had no *Candida* infection [13]. *Candida* infections were isolated more often in late-onset peritonitis and were associated with multidrug-resistant bacteria. Another study demonstrated that *C. albicans* was cultured in 11/18 cases in a bariatric surgery group and in 23/46 cases in a conventional surgery group, whereas *C. glabrata* was cultured in 13/46 cases in the conventional surgery group and 3/6 cases were resistant to fluconazole [14]. A recent, prospective, single-centre, population-based study in a surgical ICU setting observed that IAC was a frequent form of invasive candidiasis (13/22 episodes in 1149 patients) and that antifungal therapy and good source control led to a good outcome. *C. albicans* and *C. parapsilosis* were the most common invasive species, and resistance to fluconazole and itraconazole was noted in 3/22 (13.6%) cases [15]. To our knowledge, no studies have been published assessing the efficacy of antifungal agents in patients with microbiologically documented IAC.

Among the echinocandin class of antifungal agents, anidulafungin is a cyclic lipopeptide approved for the treatment of candidaemia and other forms of invasive candidiasis such as intra-abdominal abscesses and peritonitis. In the pivotal study of anidulafungin [16], there were only a few patients with microbiologically confirmed *Candida* deep-seated tissue infections. To better understand the efficacy and safety of treatment with anidulafungin in these patients, data from five prospective studies were pooled and analysed. Here, we present the results of treatment with anidulafungin in the group of patients with microbiologically documented IAC.

## Materials and methods

### Ethics

All primary studies were conducted in compliance with the Declaration of Helsinki and Good Clinical Practice Guidelines established by the International Conference on Harmonization. The final protocols, amendments and informed consent documentation were reviewed and approved by the Institutional Review Boards and the Independent Ethics Committees of the investigational centres. All patients provided written informed consent.

### Study design and treatment

Patient-level efficacy and safety data were retrospectively pooled from four open-label, non-comparative studies [17–20] and one comparative study (Pfizer data on file) with the aim of analysing the safety and efficacy of anidulafungin in the treatment of invasive candidiasis in adult patients. The comparative study was a double-blind, double-dummy, randomised, multicentre trial comparing anidulafungin with caspofungin in patients with microbiologically confirmed deep-seated tissue infection due to *Candida* species. All the studies had similar protocols and endpoints, and investigated the use of anidulafungin in a broad range of patients, including patients in the ICU and patients with neutropenia (absolute neutrophil count [ANC] < 500 cells/mm^3^) with microbiologically confirmed *Candida* intra-abdominal infections (Table 1). All studies were registered with ClinicalTrials.gov (NCT00496197, NCT00548262, NCT00537329, NCT00689338, and NCT00805740).

**Table 1:** Prospective clinical studies included in the pooled analysis (intent-to-treat population^a^)

Study	Region	Type of study	Indication	References
A8851011 (NCT00496197)^b^	USA and Korea	Open-label	Candidaemia and invasive candidiasis	Vazquez et al. 2014 [17]
A8851015 (NCT00548262)^b^	Latin America	Open-label	Candidaemia and invasive candidiasis	Nucci et al. 2014 [18]
A8851016 (NCT00537329)^b^	Asia	Open-label	Candidaemia	Mootsikapun et al. 2013 [19]
A8851019 (NCT00689338)^c^	Europe and Canada	Open-label	Candidaemia and invasive candidiasis	Ruhnke et al. 2012 [20]
A8851022 (NCT00805740)	USA, Canada, Europe, Russia, Switzerland	Double-blind, randomised	*Candida* deep-seated tissue infection	Pfizer data on file

^a^All patients who received at least one dose of anidulafungin

^b^Switch to an oral azole (fluconazole or voriconazole) was permitted after ≥ 5 days of intravenous treatment^c^Switch to an oral azole (fluconazole or voriconazole) was permitted after ≥ 10 days of intravenous treatment

Patients initiated the treatment with a 200-mg loading dose of intravenous (IV) anidulafungin on day 1 of the study, followed by a maintenance dose of 100 mg IV once daily. In all but one of the studies (A8851022 [NCT00805740]), patients could be switched to an oral azole after ≥ 5 days (A8851011 [NCT00496197], A8851015 [NCT00548262], A8851016 [NCT00537329]) or ≥ 10 days (A8851019 [NCT00689338]) of IV treatment, according to pre-specified criteria in each protocol. Antifungal treatment (IV anidulafungin plus subsequent oral azole if used) was maintained for ≥ 14 days after the last positive *Candida* culture and resolution of symptoms.

### Patients—criteria for the pooled analysis

The pooled analysis included male or female patients aged ≥ 18 years with culture-confirmed IAC from a culture specimen obtained within the preceding 96 h from a normally sterile site or newly placed drain. Patients could enter the studies based on microbiological evidence suggestive of *Candida* infection (e.g. positive blood culture for yeast). However, confirmation of *Candida* species was required within 96 h to remain in the study. Patients were also required to have more than one of the following clinical signs and symptoms of fungal infection: fever, defined as an oral or tympanic temperature > 38 °C; hypotension, defined as systolic blood pressure < 100 mg Hg or a decrease in systolic blood pressure of > 30 mmHg from baseline; and clinical signs of localised or generalised peritonitis including the presence of intra-abdominal abscess or purulent fluid from drains. Patients who had received > 48 h of prior antifungal therapy had the presence of confirmed or suspected *Candida* osteomyelitis, endocarditis or meningitis, had prosthetic devices at infection sites that could not be removed within 24 h of study entry or who had previously failed treatment for the current episode of *Candida* infection were excluded. From the pooled database, only patients with peritoneal fluid or hepatobiliary tract as sites of infection, as reported by the investigators in the case report form (CRF), were included in this analysis.

### Study endpoints

The primary endpoint of this analysis was the successful global response at the end of IV treatment (EOIVT), based on the modified intent-to-treat (MITT) population. The MITT population included all patients who received ≥ 1 dose of anidulafungin (intent-to-treat population) and who had a confirmed diagnosis of invasive candidiasis/candidaemia and a positive culture for *Candida* species within 96 h of study entry. A global response was considered successful if patients achieved both clinical success (defined as the resolution of signs and symptoms of *Candida* infection and no additional systemic or oral antifungal therapy required) and microbiological success (defined as eradication or presumed eradication of *Candida* species present at baseline).

Secondary endpoints of this analysis included the global response at the end of all therapy (EOT) in the MITT population, and the rate of all-cause mortality at day 14 and day 28 after initiation of IV therapy in the MITT populations. Treatment-emergent adverse events (TEAEs) and serious adverse events (SAEs) of all causalities in the MITT population were recorded in all studies and were summarised by Medical Dictionary for Regulatory Activities preferred term.

### Statistical analysis

Efficacy and safety analyses were for descriptive purposes, and no hypotheses for efficacy endpoints were tested. Evaluation of the data comprised primarily of summary descriptive statistics. Success rates for global response were estimated with exact 95% confidence intervals (CI) for binomial proportion (Clopper–Pearson method). In efficacy analyses, indeterminate or missing was considered failure.

Multivariate logistic regression was used to calculate odds ratios and 95% CI to identify factors that were significantly related to treatment failure in the MITT population. Risk factors for developing candidiasis were included in the protocols of all studies. Available patient characteristics data included in the multivariate analysis were age, baseline Acute Physiology and Chronic Health Evaluation (APACHE) II score, body mass index and baseline *Candida* species (mono- vs polypathogenic infections).

## Results

### Patients

The 5 studies, summarised in Table 1, included 129 anidulafungin-treated patients with deep-seated tissue infection (the MITT population). Of these, 79 patients had microbiologically confirmed intra-abdominal infection with *Candida* spp. isolated from peritoneal fluid or hepatobiliary tract. Baseline characteristics are summarised in Table 2. Neutropenic status was recorded by the investigators for 38 patients only (38/79; 48.1%). Thirty-five of the 38 patients had an ANC > 500 cells/mm^3^ (35/38; 92.1%) and 3 patients (7.9%) had an ANC ≤ 500 cells/mm^3^. The mean APACHE II score was 15.8, with 15 patients (19%) having a score > 20.

**Table 2:** Patient characteristics at baseline (*n* = 79; MITT population)

Characteristic	Mean
Age, years (SD)	60.0 (16.4)
Gender (male/female), *n*	42/37
Race, *n* (%)	
White Black Asian Other/unspecified	65 (82.3) 4 (5.1) 1 (1.3) 9 (11.4)
Weight, kg (SD)	77.3 (23.0)
Height, cm (SD)	168.1 (9.8)
APACHE II score (SD)	15.8 (6.2)
No. of patients (%) with score:	
≤ 20 > 20	64 (81.0) 15 (19.0)
Main baseline pathogen, *n* (%)^a^	
*C. albicans* *C. glabrata* *C. krusei* *C. parapsilosis* *C. tropicalis* *Candida* spp.	57 (72.2) 26 (32.9) 5 (6.3) 2 (2.5) 6 (7.6) 1 (1.3)
Site of infection, *n* (%)	
Peritoneal cavity Peritoneal cavity plus blood Peritoneal cavity plus other sites^b^ Hepatobiliary	70 (88.6) 10 (12.7) 3 (3.8) 9 (11.4)
Risk factors for invasive candidiasis, *n* (%)	
Use of broad-spectrum antibiotics Surgery^c^ Use of central venous catheter Mechanical ventilation Length of ICU stay (> 4 days) Total parenteral nutrition Renal insufficiency^d^/failure/dialysis Use of systemic steroids/immunosuppressives Solid organ transplant Chemotherapy Neutropenia^e^ Other	61 (85.9) 60 (84.5) 54 (76.1) 40 (56.3) 37 (52.1) 35 (49.3) 21 (29.6) 13 (18.3) 6 (8.5) 5 (7.0) 2 (2.8) 22 (31.0)

*ANC* absolute neutrophil count, *APACHE* Acute Physiology and Chronic Health Evaluation, *CRF* case report form, *ICU* intensive care unit, *MITT* modified intent to treat, *SD* standard deviation

^a^A single patient may have had more than one pathogen; the total percentages therefore add up to > 100%

^b^Peritoneal cavity plus abdomen; peritoneal cavity plus other; peritoneal cavity plus pleural cavity plus other

^c^Any surgical intervention (central venous catheter, drainage and abdominal surgery)

^d^Any severity of renal insufficiency at baseline was included

^e^Two patients with neutropenia as a risk factor at baseline, as reported by the investigator in the CRF, which did not necessarily correspond to the 3 patients for whom ANC was recorded in the CRF

The most frequent baseline pathogens (Table 2) were *C. albicans* (72.2%) and *C. glabrata* (32.9%), although patients could have more than one pathogen. In vitro minimum inhibitory concentration data for anidulafungin, along with susceptibility to anidulafungin, fluconazole and voriconazole, are shown in Online Resource 1. The peritoneal cavity (70/79; 88.6%) was the most common site of infection. Among the nine patients (9/79; 11.4%) with infections in the hepatobiliary tract, two had post-liver transplant complications and seven had biliary infections following liver or biliary tract interventions for malignancies. Ten patients (10/79; 12.7%) also had candidaemia, and three patients had more than one site of infection (peritoneal cavity plus abdomen; peritoneal cavity plus other; peritoneal cavity plus pleural cavity plus other)*.* Eight patients (two hepatobiliary, six peritoneal cavity) had concomitant bacterial infections with bacteria isolated from microbiological culture and reported in the CRF. However, almost all the patients (77/79) were also receiving systemic concomitant antibiotic treatment.

In the MITT population, frequent risk factors for invasive candidiasis included the use of broad-spectrum antibiotics (85.9%), surgery (84.5%), central venous lines (76.1%), and mechanical ventilation (56.3%) (Table 2).

The median duration of IV anidulafungin therapy for all patients with IAC was 14.0 days (range 1–42). In the studies where a switch from IV to oral azole therapy was permitted (all except A8851022), the median duration of therapy (IV and oral) was 16.5 days (range 1–56). In total, 26 patients switched to oral azole therapy after a median of 11.5 days (range 6–34); in studies that permitted switch after ≥ 5 days, 14 patients switched to oral therapy (median 7.0 days [range 5–21]), and in studies that permitted switch after ≥ 10 days, 12 patients switched (median 11.0 days [range 10–43]).

### Outcomes

In the MITT population, the overall global response rate (GRR) was 73.4% at EOIVT and 67.1% at EOT, as shown in Table 3. The GRRs at EOIVT and EOT by most frequent pathogens were 73.7% and 68.4% for *C. albicans*, and 76.9% and 73.1% for *C. glabrata* (Table 3).

**Table 3:** Anidulafungin GRRs (clinical and microbiological) and all-cause mortality (MITT population)

Outcome	EOIVT	95% CI	EOT	95% CI
GRR success, *n* (%)	58/79 (73.4)	63.7–83.2	53/79 (67.1)	56.7–77.5
GRR by baseline pathogen^a^, *n* (%)				
*C. albicans* *C. glabrata* *C. krusei* *C. parapsilosis* *C. tropicalis* *Candida* spp. *Candida* (unspecified)	42/57 (73.7) 20/26 (76.9) 4/5 (80.0) 1/2 (50.0) 3/6 (50.0) 1/1 (100.0) 1/1 (100)	62.3–85.1 60.7–93.1 44.9–100.0 0.0–100.0 10.0–90.0	39/57 (68.4) 19/26 (73.1) 4/5 (80.0) 1/2 (50.0) 2/6 (33.3) 1/1 (100.0) 1/1 (100)	56.4–80.5 56.0–90.1 44.9–100.0 0.0–100.0 0.0–71.1
GRR by site of infection, *n* (%)				
Hepatobiliary Peritoneal cavity Peritoneal cavity plus blood	7/9 (77.8) 42/57 (73.7) 7/10 (70.0)	50.6–100.0 62.3–85.1 41.6–98.4	6/9 (66.7) 38/57 (66.7) 7/10 (70.0)	35.9–97.5 54.4–78.9 41.6–98.4
Peritoneal cavity plus other sites^b^	2/3 (66.7)	13.3–100.0	2/3 (66.7)	13.3–100.0
All-cause mortality (no. of deaths)	5/79 (6.3)		6/79 (7.6)	

*CI* confidence interval, *EOT* end of therapy, *EOIVT* end of intravenous therapy, *GRR* global response rate; *MITT* modified intent to treat

^a^Main pathogens at baseline

^b^Peritoneal cavity plus abdomen; peritoneal cavity plus other; peritoneal cavity plus pleural cavity plus other

The GRRs at EOIVT and EOT were 77.8% and 66.7% for hepatobiliary infections and 73.7% and 66.7% for peritoneal cavity infections, respectively. The GRRs at EOIVT in patients with infections caused by a single or multiple *Candida* isolates were 74.6% (50/67) and 66.7% (8/12), respectively.

All-cause mortality at EOIVT and EOT is shown in Table 3. All-cause mortality at day 14 and day 28 was 17.7% (14/79) and 24.1% (19/79), respectively, in the MITT population. Due to limited data for neutropenia, sub-analysis was not possible.

Multivariate logistic regression was used to identify factors that were significantly related to failure. For the analysis, age, body mass index, baseline APACHE II score and baseline *Candida* species (infections caused by single vs multiple *Candida* species) were evaluated. None of the assessed factors were associated with failure.

### Safety and tolerability

The incidence and severity of TEAEs during anidulafungin IV treatment are shown in Table 4. Anidulafungin was well tolerated in this population, and most adverse events were mild or moderate in severity. SAEs were noted in 39 patients (49.4%), with the most frequent being septic shock (*n* = 8), multipleorgan dysfunction syndrome (*n* = 3), cardiac arrest (*n* = 3), hypotension (*n* = 3) and haemorrhagic shock (*n* = 3).

**Table 4:** Incidence and severity of TEAEs by system organ class during intravenous treatment with anidulafungin (MedDRA preferred terms > 2% by system organ class)

Category	*n* (%)	Mild	Moderate	Severe
Any AE	11 (13.9)	8	3	0
Blood and lymphatic system Anaemia Thrombocytopenia	2 (2.5) 1 (1.3) 1 (1.3)	1 1 0	1 0 1	0 0 0
Cardiac Cardiac failure congestive Myocardial ischaemia Supraventricular tachycardia Tachycardia	2 (2.5) 1 (1.3) 1 (1.3) 1 (1.3) 1 (1.3)	2 1 1 1 1	0 0 0 0 0	0 0 0 0 0
Gastrointestinal Abdominal pain Diarrhoea	2 (2.5) 1 (1.3) 1 (1.3)	2 1 1	0 0 0	0 0 0
Investigations Aspartate aminotransferase ↑ Blood alkaline phosphatase ↑ Immunosuppressant drug level ↑	5 (6.3) 1 (1.3) 3 (3.8) 1 (1.3)	4 1 3 0	1 0 0 1	0 0 0 0
Nervous system Headache Paraesthesia	2 (2.5) 1 (1.3) 1 (1.3)	2 1 1	0 0 0	0 0 0
Skin and subcutaneous tissue Pruritus Rash	2 (2.5) 1 (1.3) 1 (1.3)	2 1 1	0 0 0	0 0 0
Vascular Hypertension Hypotension	2 (2.5) 1 (1.3) 1 (1.3)	1 1 0	1 0 1	0 0 0

*AE* adverse event, *MedDRA* Medical Dictionary for Regulatory Activities, *TEAE* treatment-emergent adverse event

## Discussion

To our knowledge, this study represents the largest collection of patients treated with anidulafungin in microbiologically documented intra-abdominal infections in a clinical trial setting. The pooled analysis demonstrates that in the population of surgical patients with IAC treated with anidulafungin, the GRR was similar to that in the anidulafungin registrational trial [16]. The low incidence of candidaemia (12.7%) was comparable with that in a recent epidemiological study [5]. Our findings are also consistent with other investigations [5, 9], in terms of *C. albicans* (72.2%) and *C. glabrata* (32.9%) being the most common pathogens causing IAC at baseline. However, *C. parapsilosis* was not as common in this study (only 2 isolates at baseline), despite being one of the most common pathogens in another investigation (22.7%) [15]. In this pooled analysis, all *Candida* species were susceptible to anidulafungin, 84.2% were susceptible to fluconazole and 94.7% were susceptible to voriconazole. The superior susceptibility of anidulafungin over fluconazole was consistent with a study in a murine model of candidiasis [21].

Our study confirmed that abdominal surgery and use of broad-spectrum antibiotics are the most important risk factors associated with intra-abdominal invasive candidiasis.

Interestingly, in this study only 8 out of 79 patients had bacteria isolated from microbiological culture and recorded in the CRF. However, almost all patients (77/79) were receiving systemic antibiotic treatments, indicating concomitant bacterial infections, although it was not possible to know more about the sites of bacterial infection as this information was not collected in the CRF.

The median duration of IV anidulafungin in patients with intra-abdominal infections was 14 days, which was longer than the 10 days of IV anidulafungin duration described in the pooled analysis of the overall general population [22]. In the real-world AmarCAND2 study, the median duration of antifungal therapy was 14 days for targeted therapy and 17 days for empirical therapy [11]. Furthermore, in our population, a lower percentage of the patients (22% and 19%; data not shown) switched to oral therapy compared to that found by Kullberg et al. [22], where 51.8% and 34.8% of patients switched when permitted after ≥ 5 or ≥ 10 days, respectively.

Across the five studies, anidulafungin treatment was well tolerated, with most TEAEs mild or moderate in severity, consistent with the known safety profile of anidulafungin demonstrated since the registrational trials.

The anidulafungin registration trial included few patients with IAC. The pooled analysis of anidulafungin studies included 129 patients with deep-seated tissue infection [22], of whom 79 in the present study had confirmed IAC from peritoneal fluid and hepatobiliary tract. In general, other studies with echinocandins have included low numbers of patients with IAC. In one investigation where 16 patients had *Candida* peritonitis, the success rate was 87.5% (7/8) with amphotericin B and 100% (8/8) for caspofungin [23]. Success rates of 72.7% with liposomal amphotericin B and 82.4% with micafungin were observed in 28 patients with invasive candidiasis in the peritoneum [24]. In a study of 18 patients with *Candida* peritonitis, a 40% success rate was seen with caspofungin compared with 66.7% and 57.1% with micafungin 100 mg and 150 mg, respectively [25]. In a prospective study of 279 adult patients in the ICU, *C. albicans* was involved in 67% and *C. glabrata* in 15.6% [11]. Of the 179 *Candida* strains cultured from peritoneal samples, *C. albicans* was cultured in 61/96 (64%) isolates in empirically treated patients and in 59/83 (71%) receiving targeted therapy. Of the 179 empiric and targeted patients, 3 had candidaemia. Risk factors that influenced a higher mortality in the AmarCAND2 study included healthcare-associated *Candida* peritonitis, Sequential Organ Failure Assessment score ≥ 8 at ICU admission and Simplified Acute Physiologic Score II ≥ 45 at systemic antifungal treatment initiation [11]. Findings from the current analysis are similar but with lower mortality.

Intra-abdominal candidiasis is still poorly understood compared with candidaemia. To date, data and studies on the efficacy of echinocandins in intra-abdominal invasive candidiasis are scarce and, although IAC is still burdened by high mortality rates, all current international guidelines mainly address candidaemia. Source control is of paramount importance in the treatment of IAC, but adequate antifungal therapy is essential to eradicate the pathogen and to treat candidaemia, when present. For this reason, guidelines recommend an echinocandin as the drug of choice [26–28], although the Infectious Diseases Society of America does not include guidance on duration of therapy [26].

The concept of early adequate systemic antifungal therapy in patients with IAC is a point of debate based on the assumption that delayed initiation of antifungal therapy is associated with a poor outcome, as reported for patients with IAC, particularly if they also have candidaemia [29–33]. The deleterious impact of delayed initiation of systemic antifungal treatment has not yet been demonstrated for *Candida* intra-abdominal infection [5], and further evidence is needed.

A limitation of the current investigation was that four studies were open-label and were conducted in different centres within different timeframes, and yielded a small number of patients. In mitigation, all studies had similar protocols and endpoints that allowed the data to be pooled. Although two pharmacokinetic studies have demonstrated adequate anidulafungin plasma concentrations [34, 35], the lack of data to demonstrate that anidulafungin achieves adequate penetration of the peritoneal cavity may also be considered a limitation of this analysis. The analysis did not permit the assessment of whether anidulafungin would behave differently from other echinocandins in this population. Furthermore, this post hoc analysis did not include a control group and was not pre-planned by the protocols of the individual studies. It was, therefore, not possible to evaluate the adequacy of source control or of antibacterial therapy. In addition, assessment of global response and mortality in relation to the neutropenic status of patients could not be performed because data on neutropenic status were not available for all patients. Finally, only a small number of factors (age, body mass index, baseline APACHE II score and baseline *Candida* species) were evaluated by multivariate logistic regression to assess any association with failure, and none of these factors were found to be associated with failure.

This analysis of pooled data showed that the epidemiology of IAC is similar to that in previous studies, with *C. albicans* and *C. glabrata* being the most common pathogens. Also, IAC seemed to be accompanied by a lower incidence of candidaemia compared with other studies [5, 36]. Anidulafungin provided good efficacy and tolerability, which is an important consideration in critically ill surgical patients with IAC in the ICU.

Nevertheless, further studies are needed to evaluate the clinical efficacy of echinocandins, as well as their tissue penetration into the peritoneum and possibly in specific intra-abdominal districts such as bile, the liver and the pancreas.

## Electronic supplementary material

ESM 1 (DOCX 22 kb)

ESM 2 (PDF 297 kb)

ESM 3 (DOCX 22 kb)

ESM 4 (PDF 298 kb)
