# Supplementary material for: Evaluation of anidulafungin in the treatment of intra-abdominal candidiasis: a pooled analysis of patient-level data from 5 prospective studies
Source: Eur J Clin Microbiol Infect Dis. 2019 Jul 6;38(10):1849–56. doi: 10.1007/s10096-019-03617-9 (PMC6778589; doi:10.1007/s10096-019-03617-9)
Supplement: Supplementary file 3 — (DOCX 22 kb) [file 10096_2019_3617_MOESM3_ESM.docx]

Evaluation of anidulafungin in the treatment of intra-abdominal candidiasis: a pooled analysis of patient-level data from 5 prospective studies

*European Journal of Clinical Microbiology & Infectious Diseases*

Gabriele Sganga^1^ • Minggui Wang^2^ • M. Rita Capparella^3^ • Margaret Tawadrous^4^ • Jean L. Yan^5^ • Jalal A. Aram^4^ • Philippe Montravers^6^

*^1^Emergency Surgery, Fondazione Policlinico Universitario A. Gemelli IRCCS – Università Cattolica del Sacro Cuore, Roma, Italy; ^2^Fudan University, Shanghai, China; ^3^Pfizer ~~PFE~~ PIO, Paris, France; ^4^Pfizer Inc, Groton, CT, USA; ^5^Pfizer Inc, Collegeville, PA, USA; ^6^Paris Diderot Sorbonne Cite University and Bichat-Claude Bernard University Hospital, Paris, France.*

**Correspondence:** Dr. Gabriele Sganga, Emergency Surgery, Fondazione Policlinico Universitario A. Gemelli IRCCS – Università Cattolica del Sacro Cuore, Roma, Italy, Largo A. Gemelli 8, 00168 Roma, Italy.
Tel: 063015 4437/6211/4545 ([gabriele.sganga@policlinicogemelli.it](mailto:gabriele.sganga@policlinicogemelli.it))

**Online Resource 1** *In vitro* MIC data for anidulafungin, and susceptibility to anidulafungin, fluconazole and voriconazole, by *Candida* species

| **Species (*n*)** | **Anidulafungin MIC_50_ (mg/L)** | **Anidulafungin MIC_90_ (mg/L)** | **Susceptible to anidulafungin (%)** | **Susceptible to fluconazole (%)** | **Susceptible to voriconazole (%)** |
| --- | --- | --- | --- | --- | --- |
| All *Candida* species (57) | ≤0.015 | 0.03 | 100.0 | 84.2 | 94.7 |
| *C. albicans* (37) | ≤0.015 | 0.03 | 100.0 | 91.9 | 91.9 |
| *C.* *glabrata* (15) | 0.03 | 0.125 | 100.0 | 66.7 | 100.0 |
| *C.* *krusei* (1) | 0.03 | 0.03 | 100.0 | 0.0 | 100.0 |
| *C.* *tropicalis* (4) | ≤0.015 | 0.03 | 100.0 | 100.0 | 100.0 |

*MIC,* minimum inhibitory concentration
