# Supplementary material for: Evaluation of anidulafungin in the treatment of intra-abdominal candidiasis: a pooled analysis of patient-level data from 5 prospective studies
Source: Eur J Clin Microbiol Infect Dis. 2019 Jul 6;38(10):1849–56. doi: 10.1007/s10096-019-03617-9 (PMC6778589; doi:10.1007/s10096-019-03617-9)
Supplement: Supplementary file 4 — (PDF 298 kb) [file 10096_2019_3617_MOESM4_ESM.pdf]

Evaluation of anidulafungin in the treatment of intra-abdominal candidiasis: a pooled analysis of patient-level data from 5 prospective studies

European Journal of Clinical Microbiology & Infectious Diseases

Gabriele Sganga[1] • Minggui Wang[2] • M. Rita Capparella[3] • Margaret Tawadrous[4] • Jean L. Yan[5] • Jalal A. Aram[4] • Philippe Montravers[6]

[1]Emergency Surgery, Fondazione Policlinico Universitario A. Gemelli IRCCS – Università Cattolica del Sacro Cuore, Roma, Italy; [2]Fudan University, Shanghai, China; [3]Pfizer PFE PIO, Paris, France; [4]Pfizer Inc, Groton, CT, USA; [5]Pfizer Inc, Collegeville, PA, USA; [6]Paris Diderot Sorbonne Cite University and Bichat-Claude Bernard University Hospital, Paris, France.

Correspondence: Dr. Gabriele Sganga, Emergency Surgery, Fondazione Policlinico Universitario A. Gemelli IRCCS – Università Cattolica del Sacro Cuore, Roma, Italy, Largo A. Gemelli 8, 00168 Roma, Italy.

Tel: 063015 4437/6211/4545 (gabriele.sganga@policlinicogemelli.it)

Online Resource 1 *In vitro* MIC data for anidulafungin, and susceptibility to anidulafungin, fluconazole and voriconazole, by *Candida* species

**Species (*n*)**	**Anidulafungin MIC_{50} (mg/L)**	**Anidulafungin MIC_{90} (mg/L)**	**Susceptible to anidulafungin (%)**	**Susceptible to fluconazole (%)**	**Susceptible to voriconazole (%)**
All *Candida* species (57)	≤0.015	0.03	100.0	84.2	94.7
C. albicans (37)	≤0.015	0.03	100.0	91.9	91.9
C. glabrata (15)	0.03	0.125	100.0	66.7	100.0
C. krusei (1)	0.03	0.03	100.0	0.0	100.0
C. tropicalis (4)	≤0.015	0.03	100.0	100.0	100.0

MIC, minimum inhibitory concentration
